# Association between HLA-Class I and HLA-Class II Alleles and *Mycobacterium Tuberculosis* Infection in Iraqi Patients from Baghdad City

**Published:** 2014-03

**Authors:** Nidhal Abdulmohaimen Mohammed, Haitham Qassem, Farouk Hassen

**Affiliations:** 1 Department of Microbiology, Al-Nahrain College of Medicine, Al-Nahrain University, Baghdad, Iraq;; 2 Microbiologist, College of Pharmacy, Al Mustansiyria University, Baghdad, Iraq;; 3 Department of Microbiology, College of Medicine, Al Mustansiyria University, Baghdad, Iraq

**Keywords:** Tuberculosis, MHC-Class I, MHC-Class II, Lymphocytotoxicity

## Abstract

**Background:** Pulmonary tuberculosis (PT) is one of the endemic diseases in Iraq, and among the suggested predisposing factors are alleles of the human leukocyte antigen (HLA) system*. *We sought to investigate the association between HLA-class I (A and B) and -class II (DR and DQ) alleles in a sample of PT Iraqi patients.

**Methods:** lymphocytes of 105 PT patients and 40 controls were phenotyped for HLA-A, -B, -DR, and -DQ alleles by means of the microlymphocytotoxicity test using a panel of monoclonal antisera.

**Results: **HLA frequencies of B18 (16.2 vs. 2.5%; OD=7.53) and DR1 (51.4 vs. 10.0%; OD=9.53) alleles were significantly increased in the patients as compared with the controls, while B5 (6.7 vs. 25.0%), DR8 (1.9 vs. 17.5%), and DQ3 (11.4 vs. 45.0%) alleles were significantly decreased. However, a significant corrected level was maintained for only DR1, DR8, and DQ3 alleles (Pc=1.9×10^-5^, 0.02 and 1.0×10^-4^, respectively).

**Conclusion: **The results confirm the predisposing and protecting roles of HLA alleles in PT.

## Introduction

*Mycobacterium tuberculosis* is an extremely successful pathogen that has the ability to modulate the host immune response on the level of innate and acquired types.^1^^,^^2^ However, such modulation may be subjected to immunogenetic predisposition because it has been demonstrated that certain human infectious diseases occur more frequently among individuals carrying particular human leukocyte antigen (HLA) alleles.^3^ HLA-associated susceptibility to infectious disease could be due to the inability of a particular HLA protein to be associated effectively with processed antigens from the pathogen, thereby limiting the capacity of the individual to mount an effective immune response against it.^4^ Pulmonary tuberculosis (PT) is one of the infectious diseases that follow this manner. In patients with PT, positive associations have been reported between class I and II HLA alleles and the disease in different ethnic populations.^5^ In this regard, certain HLA alleles (B*40 and DQB*0301) and haplotypes (A*2-DRB1*1502) are believed to be associated with disease susceptibly,^2^^,^^5^^-^^7^ while a protective effect has also been suggested for other HLA alleles such as A*11 and B*57.^5^^,^^8^ It has been concluded accordingly that HLA plays a great role in the pathogenesis of this pathogen.^3^^,^^9^

Consequently, we aimed to study the association between HLA alleles and PT in Iraqi patients, who referred to the Institute of Tuberculosis in Baghdad city.

## Patients and Methods

*Subjects*

After obtaining approval from the Iraqi Ministry of Health’s Ethics Committee, a total of 105 Iraqi Arab patients of both genders (age range=16-63 years) were enrolled in the study. They referred to the Institute of Tuberculosis (Baghdad) for diagnosis and treatment. The diagnosis was based on clinical symptoms, X-ray chest examination, tuberculin reactivity test, and detection of acid fast bacilli by direct staining of sputum and culture.^10^ For the purposes of comparison, 40 blood donors, age-, gender-, and ethnicity-matched, were also included and considered as a control group.

*HLA Phenotyping*

Venous blood (10 ml) was drawn in a Heparinized tube, and then it was subjected to a density gradient centrifugation using lymphoprep as a separating medium to collect lymphocytes. The collected cells were further separated into T and B lymphocytes using the nylon wool method. T cells were phenotyped for HLA-class I alleles (A and B), while B cells were employed in the phenotyping of HLA-class II alleles (DR and DQ) in the microlymphocytotoxicity test,^11^ using a panel of monoclonal antibodies (Biotest Company, Germany) that were able to recognize 8 A, 20 B, 10 DR, and 4 DQ HLA antigens.

*Statistical Analysis*

Significant variations of HLA alleles between the patients and controls were assessed using the Fisher exact probability (P), and the P value was corrected for the number of antigens tested at each locus. The correction factors were 8, 20, 10, and 4 for HLA-A, -B, -DR, and -DQ loci, respectively. The results were presented in terms of observed numbers, percentage frequencies, odds ratio (OR), etiological fraction (EF), and preventive fraction (PF). The latter two estimations were calculated when the OR values were >1 (positive association) and <1 (negative association), respectively. The 95% confidence intervals (C.I.) of the OR were also given. The mathematical calculations of these estimations were carried out using the statistical package PEPI, version 4.0.

## Results

The observed numbers and percentage frequencies of HLA-class I (A and B) and -class II (DR and DQ) alleles are given in tables 1 and 2, respectively, while alleles showing significant variations between the PT patients and controls are given in table 3. As is shown in the tables, the frequencies of B18 (16.2 vs. 2.5%; OD=7.53) and DR1 (51.4 vs. 10.0%; OD=9.53) alleles were significantly increased in the patients as compared with the controls, while B5 (6.7 vs. 25.0%), DR8 (1.9 vs. 17.5%), and DQ3 (11.4 vs. 45.0%) alleles were significantly decreased. However, a significant corrected level was maintained for only DR1, DR8, and DQ3 alleles (Pc=1.9×10^ -5 ^, 0.02 and 1.0×10^ -4 ^, respectively).

**Table 1:** Observed numbers and percentage frequencies of human leukocyte antigen (HLA)-class I (A and B) alleles in the pulmonary tuberculosis patients and controls

**HLA Alleles**	**Pulmonary Tuberculosis Patients (No=105)**		**Controls** **(No=40)**		**P value**
	**No**	**%**	**No**	**%**	
A1	20	19.1	7	17.5	0.523
A2	39	37.1	11	27.5	0.001
A3	20	19.1	8	20.0	0.001
A9	22	21.0	13	32.5	0.222
A10	18	17.1	12	30.0	0.395
A11	10	9.5	3	7.5	0.613
A19	25	23.8	5	12.5	0.221
A28	2	1.9	3	7.5	----
B5	7	6.7	10	25.0	
B7	6	5.7	5	12.5	0.579
B8	23	21.9	5	12.5	0.057
B12	7	6.7	3	7.5	
B13	1	1.0	4	10.0	----
B14	3	2.9	0	0.0	----
B15	1	1.0	0	0.0	----
B16	1	1.0	0	0.0	----
B17	3	2.9	2	5.0	----
B18	17	16.2	1	2.5	0.222
B21	9	8.6	3	7.5	0.371
B22	3	2.9	4	10.0	0.371
B27	1	1.0	3	7.5	0.325
B35	29	27.6	6	15.0	----
B37	1	1.0	0	0.0	0182
B40	1	1.0	0	0.0	----
B41	11	10.5	0	0.0	----
B48	2	1.9	1	2.5	----
B53	0	0.0	4	10.0	----
B73	1	1.0	0	0.0	----

**Table 2:** Observed numbers and percentage frequencies of human leukocyte antigen (HLA)-class II (DR and DQ) alleles in the pulmonary tuberculosis patients and controls

**HLA Alleles**	**Pulmonary Tuberculosis Patients (No=105)**		**Controls** **(No=40)**		**P value**
	**No**	**%**	**No**	**%**	
DR1	54	51.4	4	10.0	0.001
DR2	28	26.7	13	32.5	0.028
DR3	30	28.6	6	15.0	0.102
DR4	28	26.7	9	22.5	0.589
DR5	11	10.5	3	7.5	0.242
DR6	6	5.7	3	7.5	0.141
DR7	14	13.3	8	20.0	0.266
DR8	2	1.9	7	17.5	0.005
DR10	4	3.8	3	7.5	0.222
DR53	6	5.7	5	12.5	0.303
DQ1	32	30.5	9	22.5	0.535
DQ2	24	22.9	8	20.0	0.898
DQ3	12	11.4	18	45.0	0.002
DQ4	9	8.6	9	22.5	0.081

**Table 3:** HLA alleles show a significant variation between the pulmonary tuberculosis patients and controls

**HLA Alleles**	**OR**	**EF or PF**	**P**	**Pc**	**95% C.I**
B5	0.21	0.20	0.004	N.S	0.064-0.69
B18	7.53	0.14	0.018	N.S	1.093-322.799
DR1	9.53	0.46	1.9×10^ -6 ^	1.9×10^-5 ^	3.056-38.922
DR8	0.09	0.16	0.002	0.02	0.009-0.522
DQ3	0.16	0.39	2.5×10^-5 ^	1.0×10^ -4 ^	0.060-0.409

OR: Odds ratio; EF: Etiological fraction; PF: Preventive fraction; P: Fisher’s exact probability; Pc: Corrected P; C.I: Confidence interval; N.S: Not significant

## Discussion

Several studies have been carried out to understand whether the susceptibility and/or immune response to *M*. *tuberculosis* is associated with HLA phenotype and/or controlled by the genes that are linked to MHC.^8^^,^^9^^,^^12^ Studies have also been conducted to find relevant T- cell epitopes of *M*. *tuberculosis* antigens and their peptides in the context of HLA-DR molecules and to define their usefulness for diagnosis or vaccine design.^13^ For HLA-class I alleles, none of the inspected alleles maintained a significant corrected variation between the patients and controls, although B8 was increased and B5 was decreased in the patients. However, other investigators have reported different positive and negative associations. Hans et al.^14^ considered A11 and B15 alleles as risk factors for tuberculosis in Americans. On the other hand, Lewinsohn et al.^15^stated that HLA-B alleles were served as the dominant MHC class I restricting molecules for anti-mycobacterium-specific CD8+ T cell responses measured in CD8+ T cells from patients with PT. These results were in agreement with the previous studies that approved the association between certain HLA types and infectivity and tuberculosis.^16^ Nevertheless, Vijaya et al.^17^ suggested that B52 (split of B5) had a negative association (protective effect) with PT, a suggestion that chimes in with the findings of the present study.

The present study also revealed that DR1 was significantly higher in the PT patients than the controls; an observation that may suggest that this allele is a PT predisposing factor in Iraqis, especially when we consider an OR of 9.53 and EF value of 0.46. In contrast, DR8 and DQ3 might be associated with a protective effect. In this regard, it has been demonstrated that an altered memory response to *M*. *tuberculosis* in DR1 negative patients was observed in favor of curing.^18^ The same authors reported that DR8 was associated with resistance to PT, as was the case in the present study. However, further inconsistent observations have also been documented. DRB1*1302 phenotype was significantly associated with PT occurring at a significantly higher allele frequency in cases by comparison with controls and especially in haplotype containing DQB1*0602/3.^19^ DQB1*0301-0304 phenotype was also significantly associated with PT, especially when it occurred in haplotype with DRB1*1101-1121.^9^ In another study, results indicated that DRB1*0803 and DQB1*0601 were found to be associated with PT disease progression, development of drug resistance, and disease severity in Koreans.^20^ In South Africa, DRB1*1302 and DQB1*0301 to -0304 were apparently associated with active PT compared to control individuals lacking these alleles.^3^ The prevalence of HLA-DRB1*0401 and HLA-DRB1*0801 was significantly decreased in Mexican patients with PT compared to their prevalence in healthy controls.^21^

## Conclusion

In Iraqi PT patients, DR1 might be considered as an indicative marker of disease susceptibility, while DR8 and DQ3 are associated with resistance against PT development. However, further studies are required to confirm these associations, and certainly a much clear picture will be gathered if HLA typing is carried out at the molecular level and with a larger number of controls. 

## References

[1] David  GR (2011). Mycobacterium tuberculosis and the intimate discourse of a chronic infection. Immunol Rev.

[2] Harriff MJ, Purdy GE, Lewinsohn DM (2012). Escape from the Phagosome: the explanation for MHC-I processing of mycobacterial antigens. Front Immunol.

[3] Lombard Z, Dalton DL, Venter PA, Williams RC, Bornman L (2006). Association of HLA-DR, -DQ, and vitamin D receptor alleles and haplotypes with tuberculosis in the Venda of South Africa. Human Immunol.

[4] Louie LG, Hartogensis WE, Jackman RP, Schultz KA, Zijenah LS, Yiu CH (2004). Mycobacterium tuberculosis/HIV-1 coinfection and disease: role of human leukocyte antigen variation. J Infect Dis.

[5] Kettaneh A, Seng L, Tiev KP, Tolédano C, Fabre B, Cabane J (2006). Human leukocyte antigens and susceptibility to tuberculosis: a meta-analysis of case-control studies. Int J Tuberc Lung Dis.

[6] Grotzke JE, Harriff MJ, Siler AC, Nolt D, Delepine J, Lewinsohn DA (2009). The mycobacterium tuberculosis phagosome is a HLA-I processing competent organelle. PLoS Pathog.

[7] Raghavan S, Selvaraj P, Swaminathan S, Alagarasu K, Narendran G, Narayanan PR (2009). Haplotype analysis of HLA-A, -B antigens and -DRB1 alleles in south Indian HIV-1-infected patients with and without pulmonary tuberculosis. Int J Immunogenet.

[8] Contini S, Pallant M, Vejbaesya S, Park M, Chierakul S, Kim S (2008). A model of phenotypic susceptibility to tuberculosis: deficient in Silico selection of mycobacterium tuberculosis epitopes by HLA alleles. Sarcoidosis vasc and diffuse lung dis.

[9] Jagannathan L, Chaturvedi M, Satish B, Satish KS, Desai A, Subbakrishna DK (2011). HLA-B57 and Gender Influence the Occurrence of tuberculosis in HIV infected people of south India. Clin Develop Immunol.

[10] Gaseitsiwe S, Valentini D, Mahdavifar S, Reilly M, Ehrnst A, Maeurer M (2010). Peptide Microarray-Based Identification of Mycobacterium tuberculosis Epitope Binding to HLA-DRB1*0101, DRB1*1501, and DRB1*0401. Clin Vaccine Immunol.

[11] Terasaki PI, MCClelland JD (1964). Micro droplet assay of human serum cytotoxine. Nature.

[12] Mizrahi V (1997). Genetic and tuberculosis, Cape Town, South Africa, 21 November. Tuber lung dis.

[13] Axelsson-Robertson R, Weichold F, Sizemore D, Wulf M, Skeiky YAW, Sadoff J (2010). HLA-B alleles served as the dominant MHC class I restricting molecules for anti-Mtb TB10.4-specific CD8+ T-cell responses measured in CD8+ T cells from patients with pulmonary TB. Immunology.

[14] Rieder HL, Cauthen GM, Comstock GW, Snider DE (1989). Epidemiology of tuberculosis in the United States. Epidemiol Rev.

[15] Lewinsohn DA, Winata E, Swarbrick GM, Tanner KE, Cook MS, Null MD (2007). Immunodominant Tuberculosis CD8 Antigens Preferentially Restricted by HLA-B. PLoS Pathog.

[16] Figueiredo JF, Rodrigues L, Deghaide NH, Donadi EA (2008). HLA profile in patients with AIDS and tuberculosis. Braz J Infect Dis.

[17] Vijaya Lakshmi, Rakh SS, Anu Radha, Hari Sai, Pantula V, Jasti S (2006). Role of HLA-B51 and HLA-B52 in susceptibility to pulmonary tuberculosis. Infect Genet Evol.

[18] Uma H, Selvaraj P, Reetha AM, Xavier T, Prabhakar R, Narayanan PR (1999). Influence of HLA-DR antigens on lymphocyte response to Mycobaterium tuberculosis culture filtrate antigens and mitogens in pulmonary tuberculosis. Tuber lung Dis.

[19] Goldfeld AE, Delgado JC, Thim S, Bozon MV, Uglialoro AM, Turbay D (1998). Association of an HLA-DQ Allele With Clinical Tuberculosis. JAMA.

[20] Kim HS, Park MH, Song EY, Park H, Kwon SY, Han SK (2005). Association of HLA-DR and HLA-DQ genes with susceptibility to pulmonary tuberculosis in Koreans: preliminary evidence of associations with drug resistance, disease severity, and disease recurrence. Hum Immunol.

[21] Amirzargar AA, Yalda A, Hajabolbaghi M, Khosravi F, Jabbari H, Rezaei N (2004). The association of HLA-DRB, DQA1, DQB1 alleles and haplotype frequency in Iranian patients with pulmonary tuberculosis. Int J Tuberc Lung Dis.

